# Supplementary material for: A Study of the Metabolic Pathways Affected by Gestational Diabetes Mellitus: Comparison with Type 2 Diabetes
Source: Diagnostics (Basel). 2022 Nov 21;12(11):2881. doi: 10.3390/diagnostics12112881 (PMC9689375; doi:10.3390/diagnostics12112881)
Supplement: Supplementary file 1 [file diagnostics-12-02881-s001.zip › diagnostics-2019981-supplementary.pdf]

Figure S1. Representative 500 MHz 1H-NMR spectrum of deproteinized serum from a pregnant woman with GDM.

1, 2-Aminobutyrate; 2, 2-Hydroxybutyrate; 3, 2-Hydroxyisovalerate; 4, 2-Oxocaproate; 5, 2-Oxoglutarate; 6, 2-Oxoisocaproate; 7, 3-Hydroxybutyrate; 8, 3-Hydroxyisobutyrate; 9, 3-Hydroxyisovalerate; 10, 3-Methyl-2-oxovalerate; 11, Acetate; 12, Acetoacetate; 13, Acetone; 14, Alanine; 15, Arginine; 16, Asparagine; 17, Aspartate; 18, Betaine; 19, Carnitine; 20, Choline; 21, Citrate; 22, Creatine; 23, Dimethylamine; 24, Formate; 25, Glutamate; 26, Glutamine; 27, Glycine; 28, Histidine; 29, Hypoxanthine; 30, Inosine; 31, Isobutyrate; 32, Isoleucine; 33, Lactate; 34, Leucine; 35, Lysine; 36, Malonate; 37, Mannose; 38, Methanol; 39, Methionine; 40, Myo-inositol; 41, N,N-Dimethylglycine; 42, O-Acetylcarnitine; 43, Ornithine; 44, Phenylalanine; 45, Proline, 46, Pyruvate; 47, Sarcosine; 48, Serine; 49, Succinate; 50, Taurine; 51, Threonine; 52, Trimethylamine N-oxide; 53, Tryptophan; 54, Tyrosine; 55, Valine; Glu, Glucose; Creat, Creatinine.

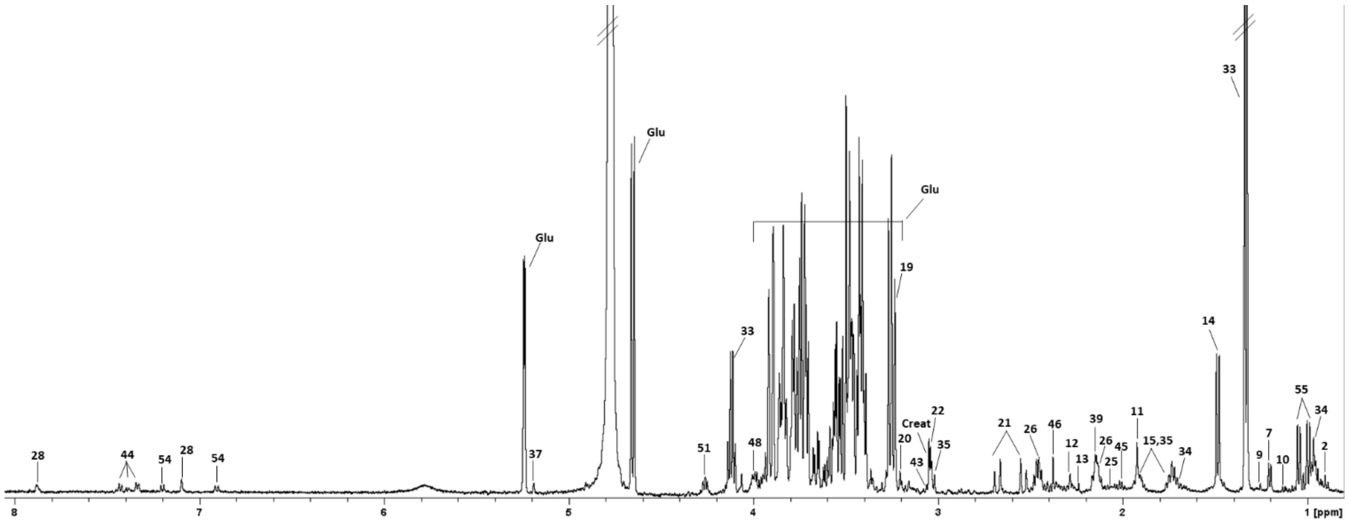

Table S1: Assignment

Metabolites	δ (H1 shift) ppm	Multiplicity	HMDB ID
2-Aminobutyrate	0,984 ; 1,904 ; 3,723	t(CH3) ; m(CH2) ; dd(CH)	(HMDB0000452)
2-Hydroxybutyrate	0,903 ; 1,641 ; 1,734 ; 4,001	t(CH3) ; m(CH2) ; m(CH2) ; dd(CH)	(HMDB0000008)
2-Hydroxyisovalerate	0,838 ; 0,961 ; 2,021 ; 3,854	d(CH3) ; d(CH3) ; m(CH) ; d(CH)	(HMDB0000407)
2-Oxocaproate	0,897 ; 1,336 ; 1,578 ; 2,733	t(CH3) ; m(CH2) ; m(CH2) ; t(CH2)	(HMDB0001864)
2-Oxoglutarate	2,428 ; 3,007	t(CH2) ; t(CH2)	(HMDB0000208)
2-Oxoisocaproate	0,941 ; 2,099 ; 2,617	d(CH3)2 ; m(CH) ; d(CH2)	(HMDB0000695)
3-Hydroxybutyrate	1,204 ; 2,313 ; 2,413 ; 4,157	d(CH3) ; m(CH2) ; m(CH2) ; m(CH)	(HMDB0000357)
3-Hydroxyisobutyrate	1,073 ; 2,491 ; 3,709	d(CH3) ; m(CH) ; m(CH2)	(HMDB0000023)
3-Hydroxyisovalerate	1,288 ; 2,366	s(CH3)2 ; s(CH2)	(HMDB0000754)
3-Methyl-2-oxovalerate	0,897 ; 1,103 ; 1,462 ; 1,704 ; 2,937	t(CH3) ; d(CH3) ; m(CH2) ; m(CH2) ; m(CH)	(HMDB0000491)
Acetate	1,924	s(CH3)	(HMDB0000042)
Acetoacetate	2,285 ; 3,454	s(CH3) ; s(CH2)	(HMDB0000060)
Acetone	2,238	s(CH3)2	(HMDB0001659)
Alanine	1,485 ; 3,794	d(CH3) ; q(CH)	(HMDB0000161)
Arginine	1,656 ; 1,916 ; 3,252 ; 3,775	m(CH2) ; m(CH2) ; t(CH2) ; t(CH)	(HMDB0000517)
Asparagine	2,863 ; 2,949 ; 4,006	m(CH2) ; m(CH2) ; dd(CH)	(HMDB0000168)
Aspartate	2,664 ; 2,805 ; 3,907	dd(CH2) ; dd(CH2) ; dd(CH)	(HMDB0000191)
Betaine	3,267 ; 3,909	s(CH3)3 ; s(CH2)	(HMDB0000043)
Carnitine	2,444 ; 3,229 ; 3,428 ; 4,576	m(CH2) ; s(CH3)3 ; m(CH2) ; m(CH)	(HMDB0000062)
Choline	3,206 ; 3,524 ; 4,073	s(CH3)3 ; dd(CH2) ; ddd(CH2)	(HMDB0000097)
Citrate	2,539 and 2,674	d(CH2) and d(CH2)	(HMDB0000094)
Creatine	3,041 ; 3,935	s(CH3) ; s(CH2)	(HMDB0000064)
Creatinine	3,052 ; 4,068	s(CH3) ; s(CH2)	(HMDB0000562)
Dimethylamine	2,715	s(CH3)2	(HMDB0000087)
Formate	8,459	s(CH)	(HMDB0000142)
Glucose	3,255 ; 3,413 ; 3,469 ; 3,545 ; 3,733 ; 3,836 ; 3,905 ; 4,655 ; 5,243	dd(CH) ; m(CH) ; m(CH) ; dd(CH) ; m(CH3) ; m(CH3) ; dd	(HMDB0000122)
Glutamate	2,059 ; 2,134 ; 2,355 ; 3,765	m(CH2) ; m(CH2) ; m(CH2) ; dd(CH)	(HMDB0000148)
Glutamine	2,146 ; 2,455 ; 3,779	m(CH2) ; m(CH2) ; t(CH)	(HMDB0000641)
Glycine	3,565	s(CH2)	(HMDB0000123)
Histidine	3,133 ; 3,239 ; 3,992 ; 7,162; 8,066	dd(CH2) ; dd(CH2) ; dd(CH) ; s(CH) ; s(CH)	(HMDB0000177)
Hypoxanthine	8,204 ; 8,224	s(CH) ; s(CH)	(HMDB0000157)
Inosine	3,845 ; 3,921 ; 4,273; 4,436 ; 6,071 ; 8,203 ; 8,321	dd(CH2) ; dd(CH2) ; dd(CH) ; dd(CH) ; d(CH) ; s(CH) ; s(	(HMDB0000195)
Isobutyrate	1,074 ; 2,392	d(CH3)2 ; m(CH)	(HMDB0001873)
Isoleucine	0,943 ; 1,014 ; 1,263 ; 1,472 ; 1,985 ; 3,679	t(CH3) ; d(CH3) ; m(CH2) ; m(CH2) ; m(CH) ; d(CH)	(HMDB0000172)
Lactate	1,333 ; 4,119	d(CH3) ; q(CH)	(HMDB0000190)
Leucine	0,965 ; 1,718 ; 3,744	t(CH3)2 ; m(CH3) ; m(CH)	(HMDB0000687)
Lysine	1,443 ; 1,728 ; 1,893 ; 3,036 ; 3,764	m(CH2) ; m(CH2) ; m(CH2) ; t(CH2) ; t(CH)	(HMDB0000182)
Malonate	3,121	s(CH2)	(HMDB0000691)
Mannose	3,389 ; 3,582 ; 3,668 ; 3,758 ; 3,797 ; 3,823 ; 3,884 ; 3,916 ; 5,192	ddd(CH) ; t(CH) ; m(CH2) ; m(CH2) ; m(CH) ; m(CH) ; dd(	(HMDB0000169)
Methanol	3,364	s(CH3)	(HMDB0001875)
Methionine	2,143 ; 2,649 ; 3,869	s(CH3)m(CH2) ; t(CH2) ; dd(CH)	(HMDB0000696)
Myo-inositol	3,289 ; 3,543 ; 3,631 ; 4,066	t(CH) ; dd(CH2) ; t(CH2) ; t(CH)	(HMDB0000211)
N,N-Dimethylglycine	2,933 ; 3,729	s(CH3)2 ; s(CH2)	(HMDB0000092)
O-Acetylcarnitine	2,149 ; 2,515 ; 2,648 ; 3,197 ; 3,611 ; 3,856 ; 5,607	s(CH3) ; dd(CH2) ; dd(CH2) ; s(CH3)3 ; dd(CH2) ; q(CH)	(HMDB0000201)
Ornithine	1,752 ; 1,841 ; 1,946 ; 3,066 ; 3,789	m(CH2) ; m(CH2) ; m(CH2) ; t(CH2) ; t(CH)	(HMDB0000214)
Phenylalanine	3,137 ; 4,002 ; 7,336 ; 7,384 ; 7,435	m(CH2) ; dd(CH) ; d(CH2) ; t(CH) ; t(CH2)	(HMDB0000159)
Proline	2,009 ; 2,069 ; 2,355 ; 3,346 ; 3,428 ; 4,139	m(CH2) ; m(CH2) ; m(CH2) ; dt(CH2) ; dt(CH2) ; dd(CH)	(HMDB0000162)
Pyruvate	2,379	s(CH3)	(HMDB0000243)
Sarcosine	2,745 ; 3,619	s(CH3) ; s(CH2)	(HMDB0000271)
Serine	3,849 ; 3,948 ; 3,965 ; 3,992 ; 4,009	dd(CH) ; d(CH2) ; d(CH2) ; d(CH2) ; d(CH2)	(HMDB0000187)
Succinate	2,411	s(CH2)2	(HMDB0000254)
Taurine	3,273 ; 3,431	t(CH2) ; t(CH2)	(HMDB0000251)
Threonine	1,333 ; 3,601 ; 4,263	d(CH3) ; d(CH) ; m(CH)	(HMDB0000167)
Trimethylamine N-oxide	3,252	s(CH3)3	(HMDB0000925)
Tryptophan	3,312 ; 3,489 ; 4,066 ; 7,218 ; 7,289 ; 7,327 ; 7,552 ; 7,741	dd(CH2) ; dd(CH2) ; dd(CH) ; t(CH) ; t(CH) ; s(CH) ; d(CH	(HMDB0000929)
Tyrosine	3,064 ; 3,204 ; 3,951 ; 6,909 ; 7,201	dd(CH2) ; dd(CH2) ; dd(CH) ; d(CH2) ; d(CH2)	(HMDB0000158)
Valine	0,995 ; 1,046 ; 2,278 ; 3,619	d(CH3) ; d(CH3) ; m(CH) ; d(CH)	(HMDB0000883)
